# Population dynamics of synanthropic rodents after a chemical and infrastructural intervention in an urban low-income community

**DOI:** 10.1038/s41598-022-14474-6

**Published:** 2022-06-16

**Authors:** Adedayo Michael Awoniyi, Cristina Venegas-Vargas, Fabio Neves Souza, Caio Graco Zeppelini, Kathryn P. Hacker, Ticiana Carvalho-Pereira, Catarina Lobo Marins, Mayara Carvalho de Santana, Arsinoê Cristina Pertile, Michael Begon, Albert I. Ko, Peter J. Diggle, Mitermayer G. Reis, James E. Childs, Eduardo Mendes da Silva, Federico Costa, Hussein Khalil

**Affiliations:** 1 Institute of Biology, Federal University of Bahia, Ondina, Salvador, 40170-115 Brazil; 2 Department of Large Animal Clinical Sciences, College Veterinary Medicine, Michigan State University, East Lansing, MI 48824 USA; 3 Centro de Pesquisas Gonçalo Moniz, Fundação Oswaldo Cruz, Ministério da Saúde, Rua Waldemar Falcão, 121, Salvador Bahia, Brazil; 4 Institute of Collective Health, Federal University of Bahia, Canela, Salvador, 40110-040 Brazil; 5 Department of Epidemiology, University of Michigan, Ann Arbor, MI 48197 USA; 6 Department of Evolution, Ecology and Behavior, University of Liverpool, Liverpool, L69 7ZB UK; 7 Department of Epidemiology of Microbial Diseases, Yale School of Public Health, New Haven, CT 06511 USA; 8 Lancaster Medical School, Lancaster University, Lancaster, LA1 4YW UK; 9 Bahia Faculty of Medicine, Federal University of Bahia, Praça Conselheiro Almeida Couto, s/n - Largo do Terreiro de Jesus, Salvador, 40025-010 Brazil; 10 National Institute of Science and Technology in Interdisciplinary and Transdisciplinary Studies in Ecology and Evolution (INCT IN-TREE), Federal University of Bahia, Salvador, Brazil; 11 Department of Wildlife, Fish and Environmental Studies (VFM), Swedish University of Agricultural Sciences (SLU), Uppsala, Sweden

**Keywords:** Animal behaviour, Ecology, Behavioural ecology, Population dynamics, Urban ecology, Bacterial infection

## Abstract

Synanthropic rodents are ubiquitous in low-income communities and pose risks for human health, as they are generally resistant to control programs. However, few or no studies have evaluated the long-term effect of chemical and infrastructural interventions on rodent population dynamics, especially in urban low-income communities, or evaluated the potential recovery of their population following interventions. We conducted a longitudinal study in a low-income community in the city of Salvador (BA, Brazil) to characterize the effect of interventions (chemical and infrastructural) on the dynamics of rodent population, and documented the post-intervention recovery of their population. We evaluated the degree of rodent infestation in 117 households/sampling points over three years (2014–2017), using tracking plates, a proxy for rodent abundance/activity. We reported a significant lower rodent activity/abundance after the chemical and infrastructural interventions (Z = −4.691 (p < 0.001)), with track plate positivity decreasing to 28% from 70% after and before interventions respectively. Therefore, the combination of chemical and infrastructural interventions significantly decreased the degree of rodent infestation in the study area. In addition, no rodent population rebound was recorded until almost a year post-intervention, and the post-intervention infestation level did not attain the pre-intervention level all through the study. Moreover, among pre-treatment conditions, access to sewer rather than the availability of food was the variable most closely associated with household rodent infestation. Our study indicates that Integrated Pest Management (IPM)-approaches are more effective in reducing rodent infestation than the use of a single method. Our findings will be useful in providing guidance for long-term rodent control programs, especially in urban low-income communities.

## Introduction

Rodents are highly adaptable animals, capable of colonizing human-altered environments, and can rapidly establish large populations where abundant resources are available, e.g. in urban low-income communities^1^. Synanthropic rodents are considered pests given their negative impacts on human health and economy^2–4^. Specifically, rats are reservoirs for several important viral, bacterial and parasitic diseases, which have likely caused more human casualties than wars^5–9^. Likewise, rodents destroy and contaminate agricultural products and infrastructure worth billions of dollars per year, while their sightings affect residents’ mental health^9^.

Urban low-income communities typically provide the environmental conditions that promote the proliferation of rodents^10–12^, which in turn complicates the management of their populations in these environments^13^. For instance, inadequate housing and sanitation (e.g. open sewers) and lack of adequate garbage collection services promote rodent presence^12^, and these are expected to worsen globally given the expected increase in the population of low-income community dwellers from the 751 million recorded in 1950 to more than 3 billion by 2050^14^. Also, it is estimated that 1 in every 4 people will either reside in informal settlements or require adequate and affordable housing by 2030^15^. Therefore, urban population increase will likely stress the local infrastructure further, especially in developing and under-developed countries, thereby leading to higher rodent proliferation and thus, more frequent human-rodent interactions.

Rodent control is one of the main public health measures implemented in urban poor communities, mainly to reduce the risk of disease transmission^3^. The methods applied are mainly chemical, using rodenticides with immediate short-term effects in the population^3,5,16^. However, several other interventions can be implemented simultaneously for longer-lasting effects. Non-chemical methods include closing of open sewer canals and regular solid waste collection^17^; hunting, trapping, capturing and other physical removal methods^18,19^; movement barriers and environmental modifications (e.g. electric fencing, pavement)^20,21^; likewise biological control using pathogens or predatory animals^22–26^. The paradigm of Integrated Pest Management (IPM) that recommends the use of several methods together has been adopted in several urban centers, with mixed results^27^.

Rodent infestations are difficult to control and/or manage, since the surviving population can rapidly recover through reproduction^28^ or immigration^6^. Following a period of population decline, rats can travel up to 90 m even in a heterogeneous urban environment^23,29,30^, and repopulate the area. Rodent control is especially difficult in urban environments due to the heterogeneity of the urban environment and because most control methods have been developed for rural or agricultural settings. Non-chemical methods also tend to have practical setbacks; for example, physical removal of rodents is time and labour-intensive, environmental modifications are expensive and labour-intensive, while electric fencing and inhibition of reproduction are non-species specific and could affect humans and other non-target species alike. As a result, lethal chemical agents remain the main method employed during rodent control programs in urban areas^31,32^. Rodenticides are somewhat effective in controlling populations three months post-intervention^10,33^. However, the long-term efficacy of a combination of chemical and infrastructural interventions on the dynamics of rodent populations, as well as the time lag between control deployment and the onset of rodent repopulation, are not clear.

Here, a case–control study was performed in Pau da Lima, an urban poor community in the periphery of Salvador, Brazil^34^. Pau da Lima consists of four valleys with similar environmental conditions that support relatively large rodent populations^11^. Three valleys (1, 2 and 3) with apparent signs of rodent infestation out of the four valleys were used for this study. The three valleys had concurrent infrastructural and chemical interventions. However, while the infrastructural intervention took place across all valleys, chemical intervention occurred only at valleys 1 and 3 (hereby referred to as the treatment valleys), while valley 2 received no chemical intervention and served as the control valley.

For the first time, we followed up with the trends in rodent infestation before and after interventions over a long-term period even in a tricky urban terrain such as Salvador, and evaluated (1) whether the combination of chemical and non-chemical interventions is indeed effective in the long-term control of rodent populations; (2) the timeline potentially required by the rodent population to return to their initial population abundance after interventions; and (3) the environmental and peri-domestic factors that are associated with rodent population rebound in the three valleys. Therefore, unlike previous studies^32,33^ that only evaluated rodent infestation before and six months after intervention, this study offers the most comprehensive longitudinal study on how rodent populations respond to chemical and non-chemical interventions in a complex urban environment such as Salvador.

## Methods

### Study area

**Figure 1:**
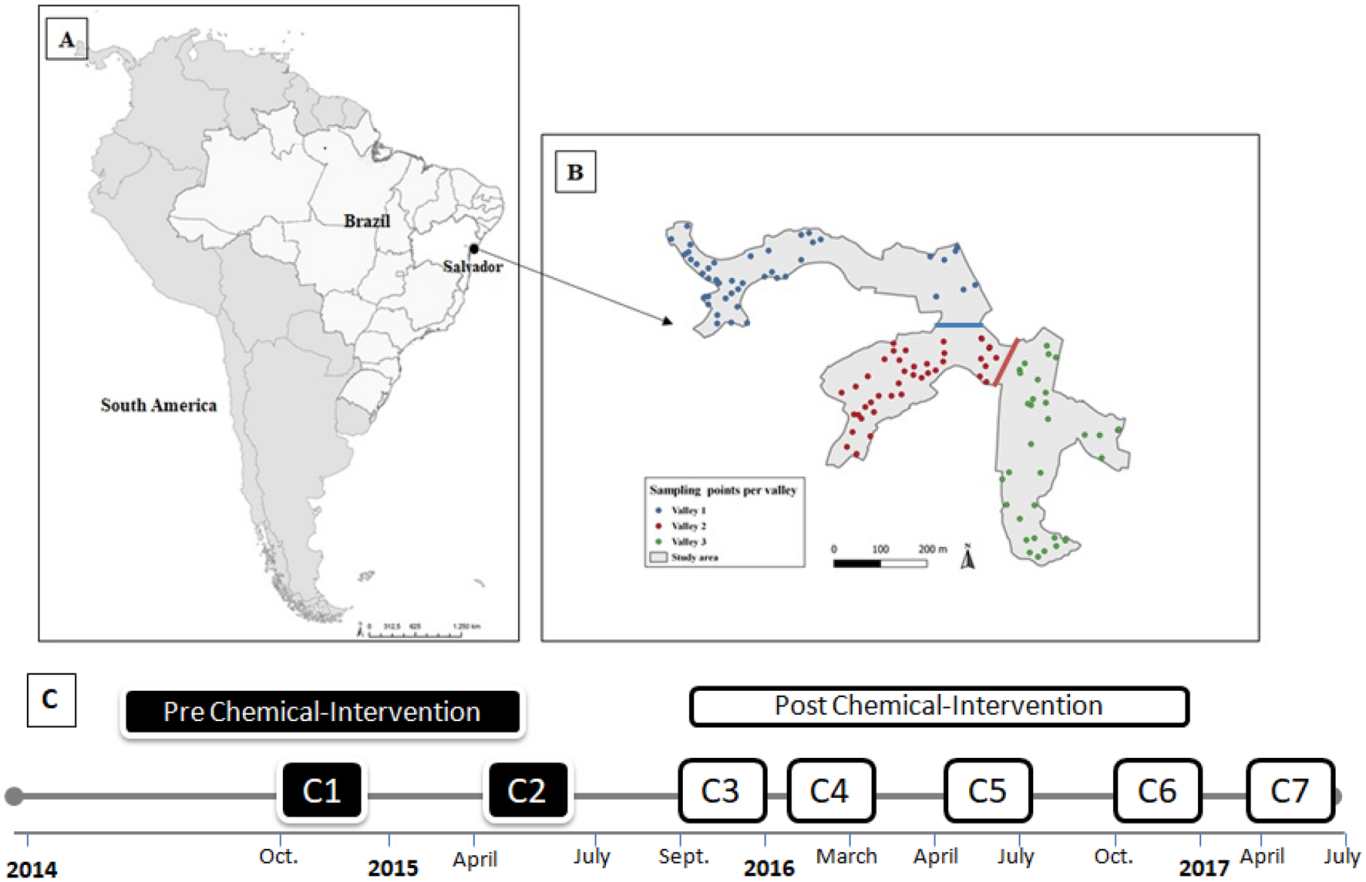
(**A**) Location of Brazil within the map of South America. (**B**) The distribution of the sampling points in the three valleys used in Pau da Lima. The figure (**A**,**B**) was generated using QGis Version 3.10 software. (**C**) Breakdown of the study timeline for rodent infestation surveys from campaign 1 to 7.

Pau da Lima (13°32′53·47″ S; 38°43′51·10″ W), is a low-income urban community (area. 0.17 km^2^) with about 128,997 inhabitants^35^. The community is characterized by inadequate housing facilities, poor basic social amenities, unsatisfactory sanitation system i.e. open sewers, improper garbage disposal and deficient garbage collection services with a history of household rodent infestation^11^. We conducted our study in three valleys (valleys 1, 2 & 3) that have been previously described by Panti-May et al.^1^ (Fig. 1A and B).

#### Sample size and frequency

One-hundred and twenty sampling points were randomly selected from the longitudinal study monitoring the urban rodents in Salvador, Brazil. However, we excluded 3 sampling points due to inaccessibility. These 117 points were randomly selected from the three valleys (Fig. 1B), with 46 sampling points from valley 1, 38 from valley 2 and 33 from valley 3, respectively.

The longitudinal sampling occurred over three years (2014–2017), and the surveys were divided into a total of 7 campaigns (“C1” through “C7”) in all valleys, with each campaign lasting 3–4 months as shown in Fig. 1C. Also, the surveys were divided into two phases, pre-intervention (C1 & C2) and chemical & infrastructural—post-intervention (C3–C7) (see Fig. 1C).

### Interventions

#### Chemical intervention

Valleys 1 and 3 served as the treatment and received chemical intervention (rodenticides), while valley 2 was considered as the control and received no chemical intervention. Using the United States’ Center for Disease Control and Prevention, CDC^36^ guidelines for interior and exterior rodent inspection, we identified, through rodent signs such as burrows, trails, or feces, active rodent spots at sampling points in both valleys 1 and 3. At each sampling point, trained staff of the Center for Control of Zoonoses (Centro de Controle de Zoonoses—CCZ) applied anticoagulant rodenticides (paraffin blocks and contact powder) within households and in peri-domestic areas in early September 2015 (i.e. first week of September), shortly before the commencement of the first post-intervention campaign (C3) which started in the second week of September through November 2015 respectively. Before the initiation of the intervention, residents were duly informed of the purpose of the study and the necessary procedures required in dealing with rodents during the study period.

Also, residents of the three valleys were reminded, during regular visits by the field team, about the importance of using appropriate waste disposal during the post intervention campaigns; putting waste out at collection points on the morning/evening of collection instead of a few days before collection; keeping pets’ food out of rodents’ reach, and the need to reduce or obstruct other sanitation-related means by which rodents could gain access to food and/or water.

The formulations used for the chemical intervention were: paraffin block (brodifacoum 0.005%) and contact powder (coumatetralyl 0.75%) respectively. Brodifacoum is a second-generation rodenticide that requires just a single dose while coumatetralyl is a first-generation rodenticide that requires multiple exposures. The paraffin blocks were applied chiefly in humid places, for example, sewers and leakages around houses, while contact powder was used in identified rodent trails and burrows that are chiefly accessible only to rodents and not non-target species.

#### Environmental/infrastructural intervention

**Figure 2:**
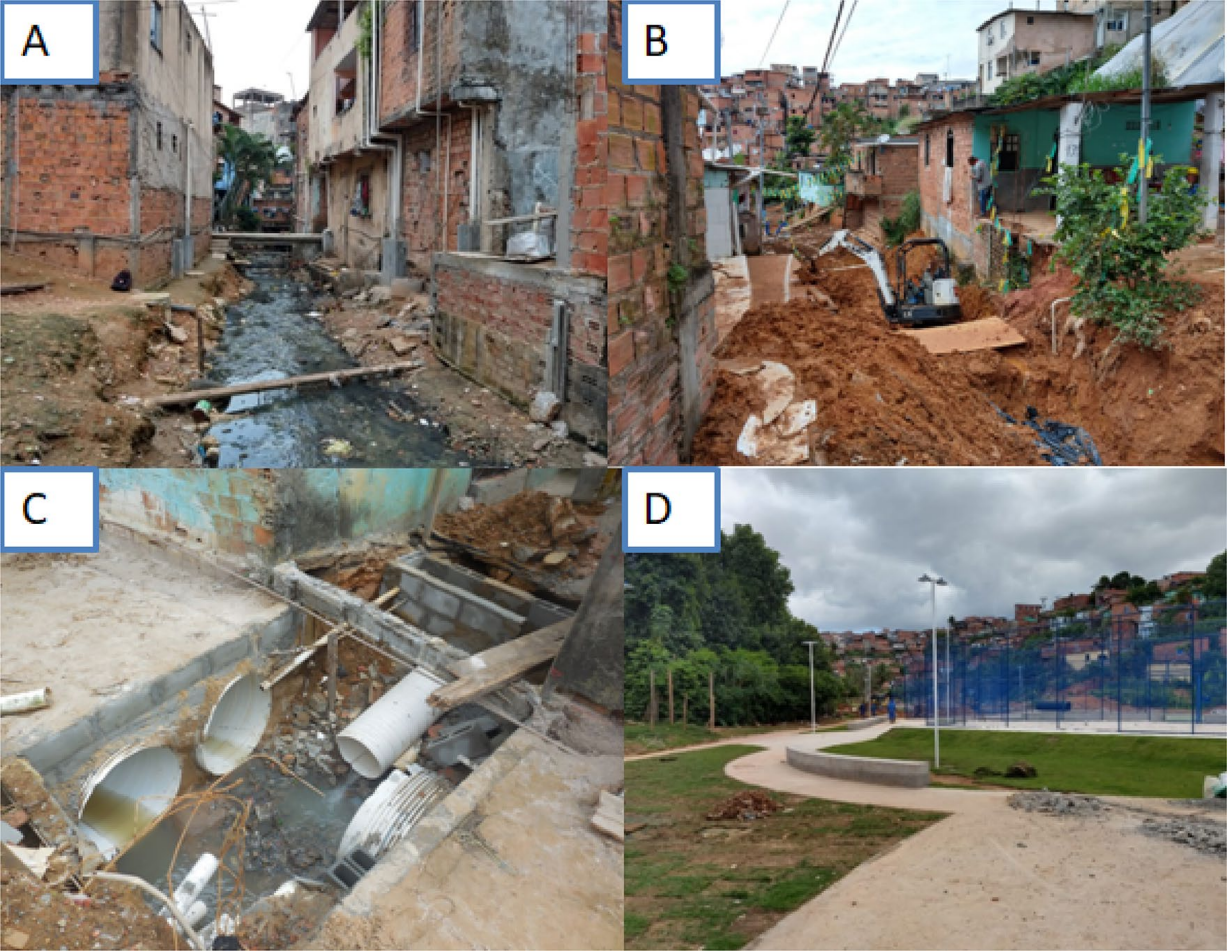
(**A**,**B**) An open sewer between households in the study area, (**C**,**D**) The study area undergoing environmental modification i.e. channeling of the drainage into a major junction, and the construction of community square, roadway and sidewalk. The figure photographed by Fabio Neves Souza, Institute of Biology, Federal University of Bahia, Brazil.

All valleys had infrastructural interventions accomplished by the city administration, that is, modification of open sewers (Fig. 2A,B and C) and the construction of public squares (common paved spaces that are free of garbage and water puddles) and pavements, both roadways and sidewalks (Fig. 2C and D), between early 2014 to the middle of 2015 which reduces rodents’ sources of food (debris from standing water) and harbourage (burrow).

### Evaluation

#### Track plates (TPs)

This method has been previously validated as a proxy for rodent infestation and comprehensively described by Hacker et al.^37^ and Eyre et al.^38^. Weather-resistant lampblack was applied to 0.2 × 0.2 m acetate sheet plates using a paint roller. The acetate plates dry off quickly (< 5 min) and allow detection of different types of marks left by rodents such as paw prints, tail marks and scratches. All TPs with any of these marks were recorded positive for rodent infestation. A total of 5 TPs were placed in a cross pattern for 2 nights at each sampling location throughout the campaigns.

The TPs were checked the morning after placement, identified by sampling location and photographed. Upon assigning 5 × 5-cell grids over each photograph, making 25 data points per plate, TPs were examined and scored by two independent trained examiners for rodent marks. Score discordances of ≥ 3 cells between examiners were flagged and reviewed to achieve a consensus.

#### Environmental evaluation

Rodents’ access to water and food sources and harbourage for rodents were recorded at each sampling point by trained CCZ personnel using a modified US’ CDC guidelines for interior and exterior rodent inspection forms^36^. Likewise, other information such as house ownership status and the presence of pets were also recorded at each sampling point.

### Statistical analysis

To evaluate the expected decline and potential rebound in rodent population after the interventions, we used generalized linear mixed effects mode (GLMMs) with logistic link and binomially distributed error structure. Our response variable at each location and campaign was coded as 1 if at least one of the TPs at a given location was positive for rodent signs, or else coded as 0. We treated valley and campaign as fixed effect factors and location as a random effect.

Before testing for a change in probability of rodent infestation (using TP positivity as a proxy) after interventions, we controlled for environmental variables that influence rodent infestation. We first used separate GLMMs to test for the relationships between the response variable and each of the following explanatory variables: presence of mud; soil & vegetation; number of domestic animals; access to garbage, water & food; proximity to sewer; and harbourage access i.e. presence of construction materials. Variables with p-values of ≤ 0.15 from the single factor models were included in a provisional multi-factor model, since opting for the more conventional level of 0.05 at this stage could fail to identify all the important variables^39^. The provisional model also included the interaction between campaign and valley to allow for any effects of interventions to vary across valleys. A mixed forward and backward stepwise model selection approach was used to determine the final model using the Akaike Information Criterion (AIC). We chose the most parsimonious model with ΔAIC < 2 compared to the minimum as the final model^40^.

Within the final model, we tested the overall significance of any intervention using the Z-statistic *C*/SE(*C*), where *C* is the contrast between pre-intervention and post-intervention campaigns, i.e. average of the estimated main effects for campaigns 1 and 2 minus the average of the estimated main effects for campaigns 3 to 7, and SE(*C*) is the standard error of *C*. Similarly, we used a “difference of differences” contrast to test whether the size of the difference between pre-intervention and post-intervention campaigns differed significantly between the chemically treated valleys, 1 and 3, and the control valley, 2.

All analyses were performed in R version 4.1.1^41^, using the lme4 (nAGQ = 9)^42^ and MuMIn (v1.43.17)^43^, ggpubr (v0.4.0)^44^, packages.

### Statement of ethical approval

While there was no direct dealing with any live animal throughout this study, as rat activity/infestation level was obtained through indirect method using track plate. However, the Ethics Committee on the Use of Animals in Research (CEUA-CPqGM) of the Fundacao Oswaldo Cruz, Centro de Pesquisas Goncalo Moniz, Salvador- Bahia, Brazil gave the approval and permission to conduct research on rats with Project Numbers: 003/2012 and 019/2016. All participating residents (interviewees) involved in the environmental aspect of the study gave their informed consent before participation, and there was no interviewee below the age of 18.

The entire study was conducted in accordance with the Brazilian laws regarding ethics in research.

## Results

### Population fluctuations across campaign per valley

**Figure 3:**
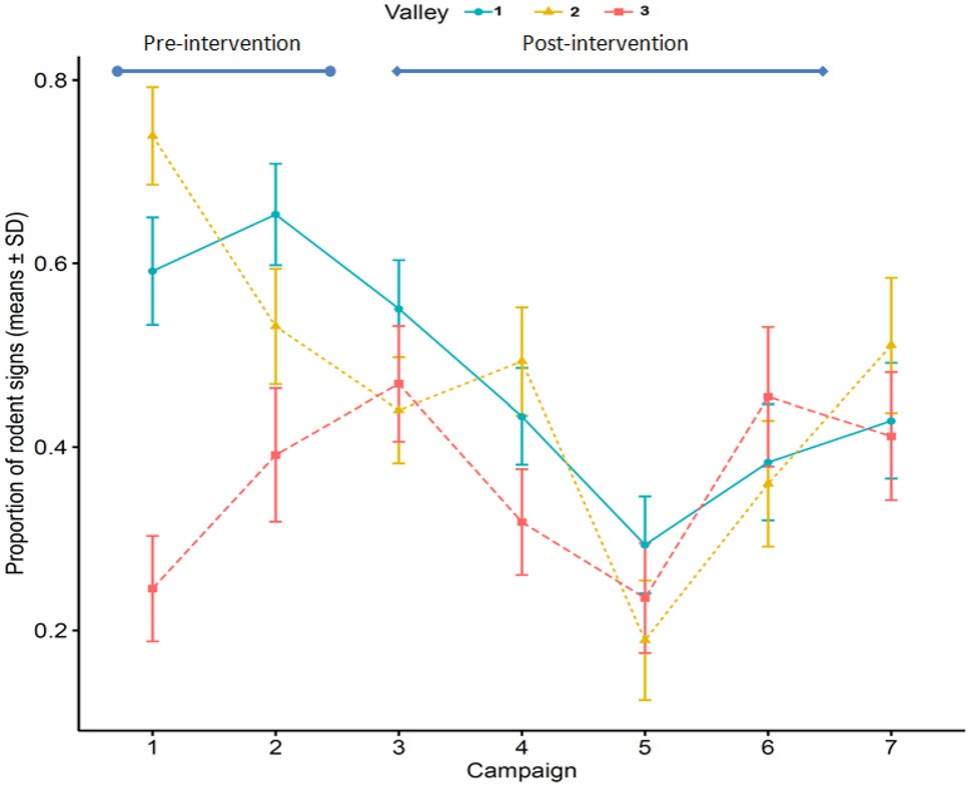
Mean with standard deviation of rodent infestation by campaign and valley (valley 1—blue, valley 2—yellow & valley 3—red colour respectively).

We recorded rodent activity across both treatments pre and post-intervention (Fig. 3). The Z-statistic to test the overall significance of any intervention was Z = −4.691 (p < 0.001) corresponding to significantly lower rat activity post-intervention. The Z-statistic to test the difference between the effects of the interventions with and without chemical treatment was Z = −2.649 (p = 0.008), corresponding to a significantly smaller pre-intervention vs post-intervention difference in the valleys that were chemically treated. All three valleys, but especially the chemically treated valleys (1 & 3) had higher signs of rodent infestation before intervention than after intervention, with mean track plate positivity decreasing from 70 to 28% before and after interventions respectively. The overall peak infestation levels were recorded during the pre-intervention campaigns (Supplemental Material 1), with a varied infestation pattern in C1 and C2, especially the sharp decline in valley 2, which was followed by a broad decline from C3 to C5 in valley 1 and 3, and a similar decline from C4 to C5 in valley 2 and then a somewhat increases from C5 to C7 across all valleys. The broad decline observed in the infestation level from C3 to C5 lasted for 10 months (Fig. 3). Also, the valley that received no chemical intervention showed the highest positivity level throughout the study (Fig. 3), while the treatment valleys (valleys that received chemical intervention) rarely exceeded 40% positivity at any given time, especially after the intervention. A GLMM with the interaction between valley and campaign indicated that C5 of valley 1 had the significantly lowest infestation level post-intervention and C6 of valley 3 had the significantly highest infestation level post-intervention in contrast to the control group (Valley 2) and the remaining campaigns (Table 1). Also, campaigns 2, 3 and 5 of valley 1, and campaigns 2 through 7 of valley 3 in relation to (valley 1 and campaign 1) had a statistically different rodent infestations level from the other observations (Table 1).

**Table 1:** Summary of the generalized linear mixed-effects model with rodent infestation associated variables, and the interaction between valley and campaign to determine the recovery rate of rodent infestation after the interventions.

Predictors	Plate positivity		
	Odds ratios	CI	p
(Intercept)	1.08	0.45–2.54	0.869
Valley [1]	0.573	0.23–1.38	0.210
Valley [3]	0.11	0.04–0.30	** < 0.001**
Campaign [2]	0.27	0.11–0.64	**0.003**
Campaign [3]	0.24	0.10–0.54	**0.001**
Campaign [4]	0.29	0.13–0.66	**0.003**
Campaign [5]	0.05	0.02–0.15	** < 0.001**
Campaign [6]	0.11	0.04–0.29	** < 0.001**
Campaign [7]	0.33	0.14–0.81	**0.016**
Access to Sewer [Yes]	1.44	1.03–2.03	**0.035**
Presence of Vegetation [Yes]	1.88	1.08–3.28	**0.025**
Presence of Mud [Yes]	1.19	0.85–1.67	0.317
Access to Garbage [Yes]	1.47	1.06–2.03	**0.022**
Valley [1] * Campaign [2]	4.89	1.54–15.49	**0.007**
Valley [3] * Campaign [2]	7.80	2.13–28.53	**0.002**
Valley [1] * Campaign [3]	3.63	1.26–10.46	**0.017**
Valley [3] * Campaign [3]	11.66	3.57–38.07	**0.001**
Valley [1] * Campaign [4]	1.56	0.54–4.52	0.416
Valley [3] * Campaign [4]	4.17	1.27–13.67	**0.018**
Valley [1] * Campaign [5]	4.62	1.20–17.80	**0.026**
Valley [3] * Campaign [5]	16.70	3.78–73.75	** < 0.001**
Valley [1] * Campaign [6]	2.70	0.79–9.17	0.112
Valley [3] * Campaign [6]	24.96	6.51–95.80	** < 0.001**
Valley [1] * Campaign [7]	1.12	0.34–3.64	0.851
Valley [3] * Campaign [7]	6.01	1.68–21.53	**0.006**
**Random effects**			
σ^2^	3.29		
τ_00 Location_	0.76		
ICC	0.19		
N_Location_	117		
Observations	1273		

Significant values are in bold.

### Factors associated with plate positivity

Seven variables (valley, campaign, treatment, presence of mud, & vegetation, access to garbage and sewers) with p-values of ≤ 0.15 from the initial analysis were considered for the generalized linear mixed-effects model (Supplemental Material 2).

The final model retained six of these seven variables (valley, Campaign, mud, vegetation, sewers and access to garbage) with an AIC of 1668.2, while the next best model had an AIC of 1669. 0 respectively. Access to sewer and garbage, and the presence of vegetation were positively associated with plate positivity-rodent infestation (Table 1). Additionally, from the Table 1, the fully treated valley- valley 3 as compared to the control valley- valley 2 showed an overall significant reduction in infestation level after interventions (OR: 0.11 [0.04–0.30], p = 0.001). Likewise, there was dissimilarity in infestation level between the treatment and control valleys before and after intervention, with valley 1 (chemical treated valley) having a significantly sustained lower infestation level during C2, C3 and C5, and valley 3 recording a significant p = 0.018 reduction in infestation level during C4 as against the control valley respectively (Table 1) which also conforms with Fig. 3.

## Discussion and conclusion

We reported a long-term decline in rodent infestation after interventions, which are different to previous studies^32,33^ that only evaluated the rodent infestation level before and after intervention via a two sampling campaigns. Here, for the first time, we were able to systematically track the continuous dynamics in urban rodent infestation for a period of three years (the most comprehensive longitudinal study to our knowledge) and then describe any associated rodent repopulation even in a complex heterogeneous urban terrain like Salvador. We reported the onset of the population rebound around 10 months post-intervention, specifically commencing after C5 across the three valleys. Also, the fully treated valleys did not return to the pre-intervention infestation levels throughout the span of the study. Therefore, the effectiveness of the interventions and the time required for the onset of rodent repopulation in our study is longer than those previously reported by de Masi et al.^32^ in São Paulo, Brazil, and Lambropoulos et al.^33^ in Baltimore, USA, who both report that rodent repopulation take around 6 months to return to pre-intervention levels, using just two campaign regimes (before and after interventions) to evaluate rodent infestation levels.

We reported high rodent infestation before intervention in the three valleys, although with a sharp pre-intervention decline in valley 2 (that is C1–C2) which could be due to rodent dispersion orchestrated by the effect of an infrastructural intervention that happened across the three valleys. Additionally, the pre-intervention decline could also be as a result of natural fluctuation in population or differences in micro-environmental attributes of the valleys more so that the decline was not sustained^2^. The high rodent infestation noticed pre-intervention might be due to the impoverished socioenvironmental conditions of the study area such as open sewage, improper garbage disposal and deficient garbage collection services and even overcrowded apartments, particularly before the interventions. These conditions have been reported to provide sufficient water, food and harbourage sources that encourage the onward proliferation of rodents in any given environment^1,11,32,45,46^.

Over the years, the main strategy used for household rodent infestation management has been the application of chemical rodenticides. Although this method is relatively easy to execute, it requires consistent investment and has a limited success-rate, with the added risk of inadvertently increasing rodents’ chances of contracting zoonoses^47^, while allowing population rebound and selective neophobia resistance among the residual population^3,48^. However, the significantly sustained reduction in rodent infestation (C2–C5 of valley 1 & C4 of valley 3) from the result of the GLMM compare to the control valley (Table 1) probably shows that the combination of a chemical and infrastructural intervention is useful in the management of rodent population especially in an heterogenous environment such as Pau da Lima. Likewise, the overall significant reduction seen in rat infestation post-intervention (Z-statistics), and reduction in the degree of infestation recorded in C4 and C5 across all valleys further suggests that interventions evaluated here may be critical in reducing rodent infestation. Nevertheless, this might not be an absolute solution to the problem, considering there was a significantly smaller reduction in plate positivity post-intervention in the chemical treated valleys, than the chemical control valley (Z-statistic for testing the difference in the effect of intervention across valleys), which might be due to varying degree of environmental confoundings across the valleys. However, the inability of the valleys to completely attain the pre-intervention infestation level after the chemical and infrastructural interventions substantiates the fact that IPM-approaches particularly here, the combination of a chemical and infrastructural intervention are more reliable in the long-term management of rodent infestation than the application of a single control method^49–51^.

Looking more closely at the combination of chemical and environmental control, a considerable reduction in rodent infestation was observed after the chemical intervention (C3 and beyond); similarly, we observed a substantial U-shaped recovery from the lowest infestation point (C5) across all the study valleys (Fig. 3). This seems to indicate that chemical intervention alone may not be sufficient for controlling rodent infestation, especially in highly rodent-infested environments. This contrasts with previous findings in the Bahamas^10^, where a reduction was observed in the rodent infestation proxy (sightings reported by locals). However, the difference in the nature of the response variables used in the studies might affect and hinder comparisons, particularly given the limitations of using rodent sightings to estimate infestation levels. In contrast, the significant reduction in rodent infestation observed in valley 2 before intervention and during infrastructural intervention -C3 may be as a result of rodent dispersion across the valley or the effectiveness of environmental modification as a component in rodent infestation management. The eventual population recovery reported in valley 2 also indicates that environmental intervention alone is as well not sufficient for controlling rodent populations in urban settings^52^. Nevertheless, it reduces the carrying capacity of the environment by denying rodents access to resources^13^.

Based on our model, in addition to the pre-treatment conditions, household accessibility to garbage and sewer had the highest association with rodent infestation level which may be as a result of the rodents being able to obtain their major source of nourishment from the sewer. Additionally, households with close proximity to sewers had higher percentage of rodent infestation (Supplemental Material 3a). Brooks^53^ simply puts that rodents flourish in this type of environment since it provides them with water and garbage that are daily thrown into the sewer.

Likewise, other factors reported as independent risk factors for rodent infestation were the presence of mud, vegetation and access to garbage (Supplemental Material 3b-d) as these provide an alternative food source for rodents^46^. These conditions are similar to those earlier reported to be positively associated with rodent infestation by Costa et al.^49^ except for the mud. Nevertheless, the pre-treatment condition of valley 3 being the lowest independent risk factor associated with rodent infestation in this study might signify that the valley has a relatively better socioeconomic condition or practice better hygiene than the other valleys, thereby lowering rodents’ source of food, water or harbourage.

Although we were able to collate practical data on rodent infestation in a longitudinal study divided over 7 campaigns for almost three years in a difficult urban environment, we were unable to survey a complete control valley (a valley without chemical and infrastructural intervention). Our original plan was to compare three independent valleys with two valleys having both chemical and infrastructural intervention and the other without any intervention. However, our original research plan was altered since the city administration decided to implement an infrastructural intervention in all the three valleys. While our result is useful in shaping the holistic impact of chemical and structural interventions both as a separate entity and a whole, further investigations using a completely negative valley (without any form of intervention) should yield a clearer result.

In conclusion, our result shows that the combination of a chemical and infrastructural intervention is a more reliable and lasting way of solving rodent problems than just the application of a single method. These findings should be useful in guiding the policymakers, non-governmental organizations, ecologists, rodent pest control organizations and others interested in rodent control on effective long-term rodent control programs. Lastly, the incorporation of this approach should assist in combating the proliferation of rodents in the urban low-income communities, while also aiding the indirect control of vector-borne zoonoses in these urban low-income environments.

## Supplementary Information

Supplementary Information.

## Data Availability

All datasets and codes used during this study are available in Zenodo under Creative Commons 4.0 license, accessible through https://doi.org/10.5281/zenodo.5796022 (Awoniyi et al., 2021).
