# Supplementary material for: Population dynamics of synanthropic rodents after a chemical and infrastructural intervention in an urban low-income community
Source: Sci Rep. 2022 Jun 16;12:10109. doi: 10.1038/s41598-022-14474-6 (PMC9203450; doi:10.1038/s41598-022-14474-6)

**Supplemental materials**

Supplemental material 1: The variation of rodent infestation (plate positivity) per valley and campaign

**
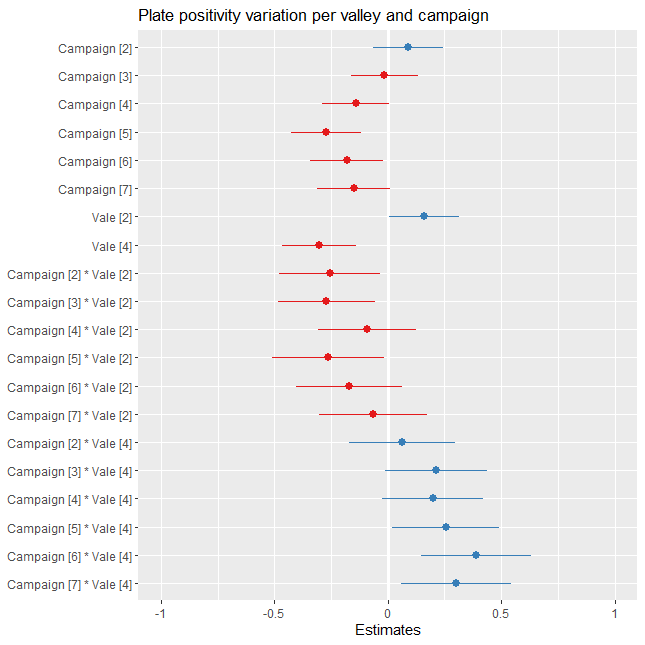
**

Supplemental material 2: Summary of the bivariate analysis for variables selection in the general model with the variables description and *p*-values

| S/N | Variable | Description of variable | p-value |
| --- | --- | --- | --- |
| 1. | Valley | Categorical, one of the valleys where the experiment was conducted | ***0.0279*** |
| 2. | Campaign/Treatment | Categorical (pre & post-intervention). Period the campaign was conducted | ***8.23e-07*** |
| 3. | Mud | Presence or absence (1 or 0). Whether there was mud ≤ 20m of the sampling point | ***0.009222*** |
| 4. | Soil | Presence or absence (1 or 0). Whether there was exposed soil ≤ 20m of the sampling point | *0.4473* |
| 5. | Vegetation | Presence or absence (1 or 0). Whether there was vegetation ≤ 20m of the sampling point | ***0.001757*** |
| 6. | Dogs | Presence or absence and counts. Whether the sampling point (household) has dogs and the number | *0.253* |
| 7. | Chicken | Presence or absence and counts. Whether the sampling point (household) has chicken and the number | *0.878* |
| 8. | Garbage | Categorical (Yes or No). Whether there was exposed garbage ≤ 20m of the sampling point | ***1.67e-05*** |
| 9. | Water | Presence or absence (1 or 0). Presence of water access ≤ 20m of the sampling point | *0.413* |
| 10. | Food | Presence or absence (1 or 0). Presence of food access ≤ 20m of the sampling point | *0.9370* |
| 11. | Sewer | Presence or absence (1 or 0). Presence of sewer access ≤ 20m of the sampling point | ***0.002351*** |
| 12. | Construction materials | Presence or absence (1 or 0). Presence of construction materials ≤ 20m of the sampling point | *0.4771* |
| 13. | Debris | Categorical (Yes or No). Presence of debris ≤ 20m of the sampling point | *0.21949* |

Supplemental material 3a-d: Prediction of rodent dynamics by the significantly correlated variables


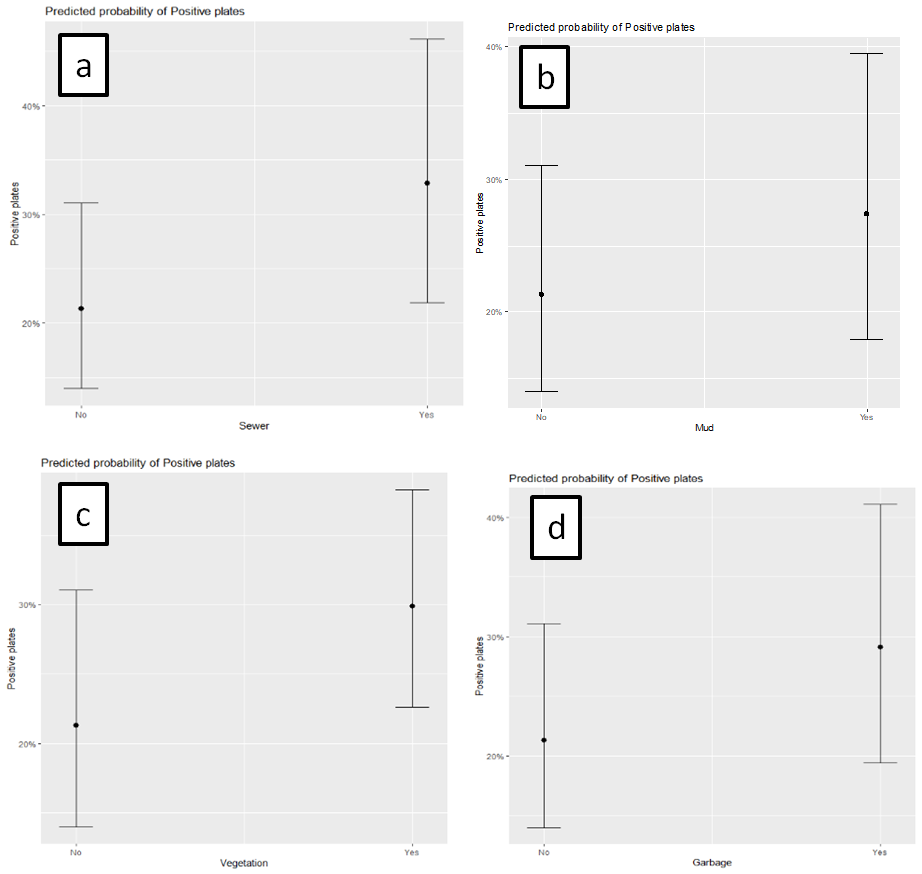

Supplement: Supplementary file 1 — Supplementary Information. [file 41598_2022_14474_MOESM1_ESM.docx]
